# Ethanol Extract of *Ulmus pumila* Root Bark Inhibits Clinically Isolated Antibiotic-Resistant Bacteria

**DOI:** 10.1155/2013/269874

**Published:** 2013-10-08

**Authors:** Yong-Ouk You, Na-Young Choi, Kang-Ju Kim

**Affiliations:** ^1^Department of Oral Biochemistry, School of Dentistry, Wonkwang University, Iksan 570-749, Republic of Korea; ^2^Wonkwang Research Institute for Food Industry, Iksan 570-749, Republic of Korea; ^3^College of Education, Wonkwang University, Iksan 570-749, Republic of Korea; ^4^Department of Oral Microbiology, School of Dentistry, Wonkwang University, Iksan 570-749, Republic of Korea

## Abstract

In this study, root bark of *Ulmus pumila* (*U. pumila*) was extracted with ethanol, and then the antimicrobial effects were tested on clinically isolated 12 MRSA strains and 1 standard MRSA strain. *U. pumila* showed antibacterial activities against all MRSA strains. Minimum inhibitory concentration (MIC) of *U. pumila* root bark against all MRSA strains revealed a range from 125 to 250 **μ**g/mL. These results may provide the scientific basis on which *U. pumila* root bark has traditionally been used against infectious diseases in Korea. In real-time PCR analysis, the sub-MIC (64–125 **μ**g/mL) concentrations of *U. pumila* root bark extract showed the inhibition of the genetic expressions of virulence factors such as *mecA*, *sea*, *agrA*, and *sarA* in standard MRSA. Phytochemical analyses of *U. pumila* root bark showed relatively strong presence of phenolics, steroids, and terpenoids. These results suggest that the ethanol extract of *U. pumila* root bark may have antibacterial activity against MRSA, which may be related to the phytochemicals such as phenolics, steroids, and terpenoids. Further studies are needed to determine the active constituents of *U. pumila* root bark responsible for such biomolecular activities.

## 1. Introduction

*Staphylococcus aureus* (*S. aureus*) is one of the most common bacteria in humans. *S. aureus* is normally present in the skin, nasal cavity, or laryngopharynx of healthy men and opportunistically causes a local or systemic infection [1, 2]. However, *S. aureus* is a causative bacteria of nosocomial infection and occupies more than 80% of pyogenic infection such as abscess and septicemia [2–5].

Penicillin was developed in 1941 and has been used as a therapeutic agent indicated for bacterial infections. Since then, numerous antibiotic agents have been developed and are effective against bacterial infections, but the appearance of antibiotic-resistant bacterial strains caused a big problem in the treatment of patients [6, 7]. The appearance of such antibiotic-resistant bacterial strains tends to increase due to the overuse of antibiotics. Antibiotic-resistant strains which became an important issue in the world include methicillin-resistant *Staphylococcus aureus *(MRSA) [8]. These bacteria strains have multidrug resistance showing resistance to various antibiotic agents such as *β*-lactams, or aminoglycosides. Treatment of patients infected with these bacterial strains is known to be very difficult [9, 10], so MRSA is one of the important causes of modern chronic infectious diseases. It is known that the resistance mechanisms of MRSA to methicillin include (1) production of *β*-lactamase, which inactivate the *β*-lactam antibiotics and (2) the possession of *mecA *gene that produces penicillin-binding proteins, such as PBP, PBP2′, or PBP2a, which have low affinity to *β*-lactam antibiotics [7]. Since MRSA shows resistance to various antibiotics, it is necessary to develop new substances for the treatment of MRSA. Several natural substances may be candidates for new antibiotic substances [11]. We have explored natural substances with antimicrobial effects on MRSA [12–14].

*Ulmus pumila *(*U. pumila*) is a natural herb that has traditionally been used for the treatment of infections in Korea. *U. pumila*, belonging to the botanical classification of Ulmaceae, is distributed in Korea, Japan, northern China, Sakhalin, and East Siberia. This tree grows to 15 meters and its bark is dark brown in color. Owing to its antibacterial and anti-inflammatory reaction, *U. pumila* has been traditionally used for abscess, infection, edema, rhinitis, empyema, and otitis media. It has also been used for gastric and duodenal ulcers as well as gastric cancer [15–17]. 

In this study, *U. pumila* was extracted with ethanol, and then the antimicrobial effects of *U. pumila* ethanol extract were tested on clinically isolated 12 MRSA strains and 1 standard MRSA strain, and phytochemical analysis was performed.

## 2. Materials and Methods

### 2.1. Plant Material and Extraction

The bark of *U. pumila *was obtained from the oriental drug store, Dae Hak Yak Kuk (Iksan, South Korea). The identity was confirmed by Dr. Bong-Seop Kil at the Department of Natural Science, Wonkwang University.

Voucher specimen (number 09-03-26) has been deposited at the Herbarium of Department of Oral Biochemistry in Wonkwang University. Dried bark of *U. pumila *(100 g) was chopped into small pieces and was extracted 2 times with 1000 mL of ethanol for 72 h at room temperature. The filtration of the extracted solution and evaporation under reduced pressure yielded ethanol extracts (9.3 g). After the extract was thoroughly dried for complete removal of solvent, the dry extract was then stored in a deep freezer (−70°C).

### 2.2. Bacterial Strains

Staphylococcal strains listed in Table 1 were 12 clinical isolates (MRSA) from Wonkwang University Hospital and the standard strain of MRSA ATCC 33591. Antibiotic susceptibility was determined from the size of the inhibition zone, in accordance with guidelines of Clinical & Laboratory Standards Institute (CLSI, 2010), and the used strains were defined as MRSA based on occurrence of the *mecA* gene and their resistance to oxacillin [18]. *β*-Lactamase activity was also determined using the DrySlide Beta Lactamase test (Difco Laboratories, Detroit, MI, USA) according to manufacturer's specification. After culturing on Mueller-Hinton agar (Difco Laboratories), the bacteria were suspended in Mueller-Hinton broth (Difco Laboratories) and used for inoculation. All MRSA strains used in this study are identified as MRSA [13].

### 2.3. Disc Diffusion Method

As the first screening, the paper disc diffusion method was used to determine antibacterial activity, which is based on the method described previously [12, 19]. Sterile paper discs (6 mm; Toyo Roshi Kaisha, Japan) were loaded with 50 *μ*L of different amounts (0.25, 0.5, and 1 mg) of the extracts dissolved in dimethyl sulfoxide (DMSO) and were left to dry for 12 h at 37°C in a sterile room. Bacterial suspensions were diluted to match the 0.5 MacFarland standard scale (approximately 1.5 × 10^8^ CFU/mL) and they were further diluted to obtain a final inoculum. After Mueller-Hinton agar was poured into Petri dishes to give a solid plate and inoculated with 100 *μ*L of suspension containing 1 × 10^8^ CFU/mL of bacteria, the discs treated with extracts were applied to Petri dishes. Ampicillin and oxacillin were used as positive controls and paper discs treated with DMSO were used as negative controls. The plates were then incubated at 35°C for 24 h in a incubator (Vision Co., Seoul, Korea). Inhibition zone diameters around each disc were measured and recorded at the end of the incubation time.

### 2.4. Determination of Minimum Inhibitory Concentrations (MICs)

MICs were determined by the agar dilution method, which is based on the method described previously [12, 20]. MICs of ampicillin and oxacillin were also determined. A final inoculum of 1 × 10^4^ CFU/mL was spotted with a multipoint inoculator (Denley Instruments, Sussex, UK) onto agar plates. The plates were then incubated at 35°C for 24 h in the incubator (Vision Co., Seoul, Korea). The MIC was defined as the lowest concentration of extracts at which no visible growth was observed. The minimum concentration of extracts that inhibited 50% and 90% of the isolates tested was defined as MIC_50_ and MIC_90_, respectively.

### 2.5. Real Time Polymerase Chain Reaction (PCR) Analysis

To determine the effect of *U. pumila* extract on gene expression, a real-time PCR assay was performed. The sub-MIC (32–125 *μ*g/mL) of *U. pumila* extract was used to treat and culture MRSA ATCC 33591 for 24 h. Total RNA was isolated from MRSA by using Trizol reagent (Gibco-BRL) according to the manufacturer's instructions and was treated with DNase to digest contaminated DNA. Then, cDNA was synthesized using a reverse transcriptase reaction (Superscript; Gibco-BRL). The DNA amplifications were carried out using an ABI-Prism 7000 Sequence Detection System with Absolute QPCR SYBR Green Mixes (Applied Bio systems Inc., Foster City, CA, USA). The primer pairs that were used in this study were described by previous reports [21–23] and are listed in Table 2. 16S rRNA was used as an internal control.

### 2.6. Phytochemical Screening

Phytochemical tests of extracts were performed as previously described [24, 25]. Mayer's reagent was used for alkaloids, ferric chloride reagent for phenolics, Molisch test for glycosides, Biuret reagent for proteins, Mg-HCl reagent for flavonoids, Liebermann-Burchard reagent for steroids, and silver nitrate reagent for organic acids.

### 2.7. Statistical Analysis

All experiments were carried out in triplicate. Data were analyzed using the statistical package for social sciences (SPSS). Differences between means of the experimental and control groups were evaluated by the Student's *t*-test.

## 3. Results

In this study, the antibiotic effect of ethanol extract of *U. pumila* on clinically isolated MRSA strain 12 and standard MRSA strain 1 (ATCC 33591) was examined. As a result of measuring antibacterial activity of *U. pumila* using the disc diffusion method, *U. pumila *showed antibacterial activities against all strains (Table 3). In all MRSA strains, 1 mg of *U. pumila* showed 14–19 mm of inhibition zone and 0.5 mg of *U. pumila *showed 9–16 mm of inhibition zone.

**Figure 1:**
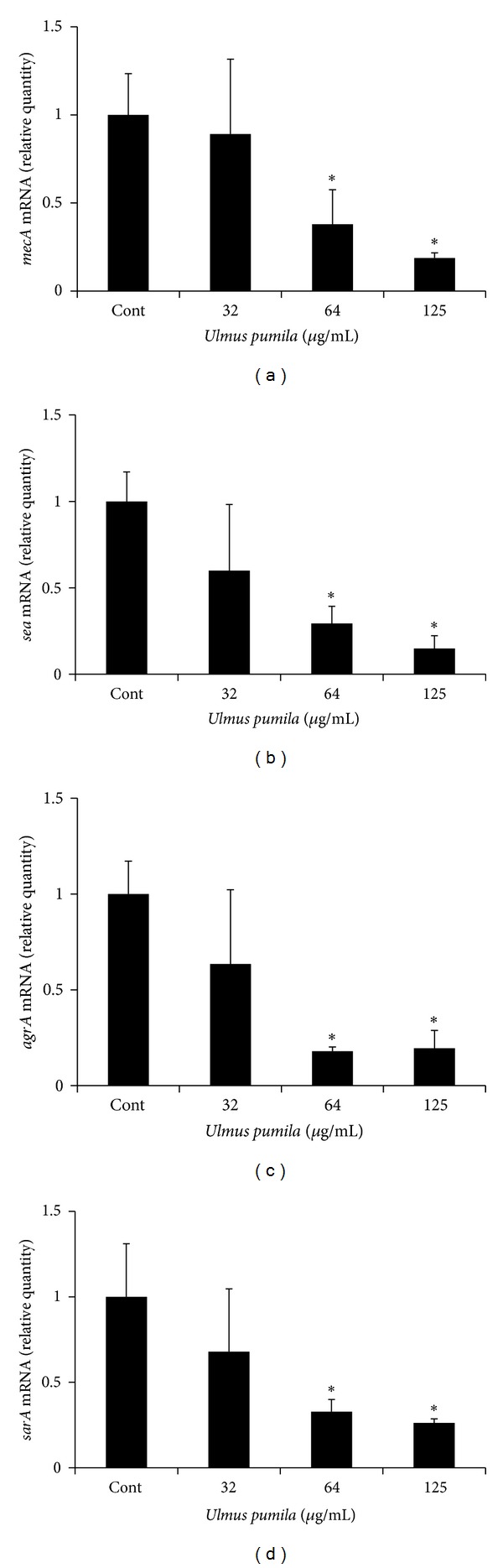
Real-time PCR analysis of expression of several virulence factor genes. MRSA ATCC 33591 was cultured and treated with sub-MIC concentrations (32–125 *μ*g/mL) of *U. pumila* extract, and real-time PCR analysis was then performed as described in the Section 2. *mecA*, *sea*, *sarA*, and* agrA *expressions were significantly inhibited at concentration higher than 64 *μ*g/mL. Each value is expressed as a mean ± standard deviation. Significance was determined at *P < 0.05 when compared with the control.

This experimental result was confirmed through MIC measurement (Table 4). Ethanol extract of *U. pumila* showed a range of MICs from 125 *μ*g/mL to 250 *μ*g/mL in all MRSA strains. In most strains, the growth of bacteria was inhibited noticeably from 250 *μ*g/mL of *U. pumila *concentration. The MICs for ampicillin and oxacillin against MRSA strains clinically isolated, which had been used as the positive control, were 4–46 *μ*g/mL and 4–16 *μ*g/mL, respectively. These results showed that the MRSA strains isolated clinically have resistance to ampicillin and oxacillin. From this experimental result, these clinically isolated MRSA strains showed resistance to ampicillin or oxacillin. We performed real-time PCR analysis to examine the effect of sub-MIC (32–125 *μ*g/mL) concentrations of *U. pumila* extract on the genetic expressions of virulence factors in standard MRSA (ATCC 33591). The expressions of *mecA*, *sea*, *agrA*, and *sarA *were significantly decreased in MRSA when it was treated with the sub-MIC (63–125 *μ*g/mL) concentrations of *U. pumila* extract (Figure 1).

As a result of phytochemical analysis of *U. pumila*, phenolics, steroids, and terpenoids were detected with a relatively high content; glycosides were detected with a medium level of content; flavonoids, peptides, and organic acids were detected with low content; but alkaloids were nearly never detected (Table 5).

## 4. Discussion

MRSA, an antibiotic-resistant strain, causes severe complex clinical problems in many parts of the world. Therefore, new agents are needed to treat the MRSA. Some natural products are candidates of new antibiotic substances. Traditionally, *U. pumila *has been used for the treatment of infectious diseases in Korea. In this study, antibacterial activities of ethanol extract of *U. pumila* on clinically isolated 12 MRSA strains and 1 standard MRSA strain were examined.

Antibacterial activities of *U. pumila* were measured by using the disc diffusion method, which were then also confirmed through MIC measurements. *U. pumila* ethanol extract showed antibacterial abilities against all the strains, 12 strains of MRSA isolated clinically and 1 standard strain of MRSA. The fact that *U. pumila *extract suppresses growth of *S. aureus *could provide the scientific basis, that the extract had been used for the treatment of infectious diseases. 

According to previous studies, *U. pumila* was known to contain steroidal chemicals such as *β*-Sitosterol, phytosterol, and stigmasterol; terpenoid chemicals such as friedelin, epifriendelalol, and taraxerol; phenolics such as tannin; and polysaccharides such as starch [16]. In this study, the phytochemical analysis of *U. pumila* showed a result of relatively high content of phenolics, steroids, and terpenoids. This result suggests that the antibacterial activity of* U. pumila* may be related with these chemicals. However, more additional researches are required to identify the antibacterial components in *U. pumila*.

Several mechanisms by which microorganisms can overcome antimicrobial agents are known. These mechanisms include production of drug insensitive enzymes, modification of targets for drug, and multidrug resistance pump which discharge the antimicrobial agents entered in bacterial cells. *mecA *is the typical multidrug resistance gene, and *fem*, *llm*, and* sigB* are also the other multidrug resistance genes [26]. Recent studies reported that some medicinal plants contain multidrug resistance inhibitor that is to lower the MIC of antimicrobial agents [27]. In the present study, the effect of sub-MIC (32–125 *μ*g/mL) concentrations of *U. pumila* extract on the genetic expression of *mecA *was determined by real-time PCR analysis. The expression of *mecA *was significantly decreased in standard MRSA (ATCC 33591) when it was treated with concentration higher than 64 *μ*g/mL. Additional investigation is necessary to determine whether *U. pumila* may have multidrug resistance inhibitors. 

A virulence factor gene, *sea*, encodes Staphylococcal enterotoxin A which is one of major virulence factors in MRSA [28]. Staphylococcal enterotoxin A is one cause of gastroenteritis in humans and acts as a superantigen. In this study, the sub-MIC concentrations of *U. pumila* extract significantly inhibited *sea* expression. *sea *gene expression in MRSA is regulated by global regulators such as *agr* and *sarA* genes [21]. In our study, sub-MIC (64–125 *μ*g/mL) concentrations of *U. pumila* extract showed the inhibition of *agrA* and* sarA* expressions in MRSA. *agrA *encodes accessory gene regulator A which positively regulates exotoxin-encoding genes. *sarA* also upregulates expression of virulence factor genes. Previous research has shown that inhibition of *agrA* or* sarA* expression by some chemicals such as thymol or clindamycin reduces transcription of exotoxin-encoding genes [21]. In the present study, suppressive effect of *U. pumila* extract on *sea *gene expression may, in part, be related with the inhibitory effect of *U. pumila* extract on *agrA* and* sarA* expressions [29].

In conclusion, *U. pumila* has antibacterial effects against MRSA, which may be related to the phytochemicals such as phenolics, steroids, or terpenoids which are highly present in *U. pumila*.

## Figures and Tables

**Table 1:** Bacterial strains used the test of antibacterial activity.

Strains	Class	*mecA* gene	*β*-Lactamase activity	Antibiotic resistance pattern
*S. aureus* ATCC 33591	MRSA	+	+	AM, OX, CF, E
Clinical isolates				
*S. aureus* (OMS 1)	MRSA	+	+	AM, OX
*S. aureus *(OMS 2)	MRSA	+	+	AM, OX, E
*S. aureus *(OMS 3)	MRSA	+	+	AM, OX, CF, E
*S. aureus* (OMS 4)	MRSA	+	+	AM, OX, E
*S. aureus* (OMS 5)	MRSA	+	+	AM, OX, CF, E
*S. aureus* (OMS 6)	MRSA	+	+	AM, OX, E
*S. aureus *(OMS 7)	MRSA	+	+	AM, OX, CF, E
*S. aureus* (OMS 8)	MRSA	+	+	AM, OX, CF, E
*S. aureus *(OMS 9)	MRSA	+	+	AM, OX, CF, E
*S. aureus *(OMS 10)	MRSA	+	−	AM, OX, E
*S. aureus* (OMS 11)	MRSA	+	+	AM, OX
*S. aureus *(OMS 12)	MRSA	+	+	AM, OX

+: positive; −: negative; AM: ampicillin; OX: oxacillin; CF: cephalothin; E: erythromycin.

OMS indicates Staphylococcal strains of Department of Oral and Maxillofacial Surgery, Wonkwang University Hospital.

**Table 2:** Nucleotide sequences of primer used for real-time PCR in this study.

Gene	Gene description	Primer sequences (5′-3′)	
*16srRNA *	Normalizing internal standard	Forward	ACTGGGATAACTTCGGGAAA
		Reverse	CGTTGCCTTGGTAAGCC
*mecA *	Penicillin binding protein 2′	Forward	GTTAGATTGGGATCATAGCGTCATT
		Reverse	TGCCTAATCTCATATGTGTTCCTGTAT
*sea *	Staphylococcal enterotoxin A	Forward	ATGGTGCTTATTATGGTTATC
		Reverse	CGTTTCCAAAGGTACTGTATT
*agrA *	Accessory gene regulator A	Forward	TGATAATCCTTATGAGGTGCTT
		Reverse	CACTGTGACTCGTAACGAAAA
*sarA *	Staphylococcal accessory regulator A	Forward	TGTTATCAATGGTCACTTATGCTG
		Reverse	TCTTTGTTTTCGCTGATGTATGTC

**Table 3:** Antimicrobial activity (mm inhibition zones diameter) of extracts of *Ulmus pumila* against 12 methicillin-resistant *Staphylococcus aureus *(MRSA) and 1 standard MRSA.

Strains	Zone of inhibition (mm)				
	*Ulmus pumila* (mg)			Ampicillin^a^	Oxacillin^b^
	0.25	0.5	1	10 *μ*g	1 *μ*g
*S. aureus *(ATCC33591)	10	12	14	10	ND
Clinical isolates					
*S. aureus* (OMS 1)	11	13	16	12	9
*S. aureus* (OMS 2)	ND	9	14	11	ND
*S. aureus* (OMS 3)	9	11	15	11	ND
*S. aureus *(OMS 4)	10	14	16	11	ND
*S. aureus* (OMS 5)	10	12	14	11	ND
*S. aureus* (OMS 6)	11	15	17	10	ND
*S. aureus* (OMS 7)	ND	10	14	8	ND
*S. aureus* (OMS 8)	9	13	15	10	ND
*S. aureus *(OMS 9)	9	12	14	10	ND
*S. aureus *(OMS 10)	12	15	18	17	ND
*S. aureus* (OMS 11)	11	14	17	11	ND
*S. aureus* (OMS 12)	13	16	19	11	ND

ND: no detected activity at this concentration; C: chloroform extract; B: *n*-butanol extract; M: methanol extract; A: aqueous extract.

^a^Ampicillin resistance ≤28 mm.

^b^Oxacillin resistance ≤10 mm.

**Table 4:** Minimal inhibitory concentrations (MICs) of the *Ulmus pumila*, ampicillin, and oxacillin against 12 methicillin-resistant *Staphylococcus aureus *(MRSA) and 1 standard MRSA.

Strains	MIC (*μ*g/mL)		
	*Ulmus pumila *	Ampicillin^1^	Oxacillin^2^
*S. aureus* ATCC 33591	250	32	256
Clinical isolates			
*S. aureus* (OMS 1)	250	32	8
*S. aureus* (OMS 2)	250	32	4
*S. aureus* (OMS 3)	250	64	4
*S. aureus* (OMS 4)	250	64	4
*S. aureus* (OMS 5)	250	32	4
*S. aureus* (OMS 6)	125	64	16
*S. aureus* (OMS 7)	250	32	16
*S. aureus* (OMS 8)	250	32	8
*S. aureus* (OMS 9)	250	64	8
*S. aureus* (OMS 10)	125	4	4
*S. aureus* (OMS 11)	125	64	16
*S. aureus* (OMS 12)	125	64	4

^1^Ampicillin resistance is an ampicillin MIC of ≥0.25 *μ*g/mL.

^2^Oxacillin resistance is an oxacillin MIC of ≥4 *μ*g/mL.

OMS indicates Staphylococcal strains of Department of Oral and Maxillofacial Surgery, Wonkwang University Hospital.

**Table 5:** Phytochemical analysis of the ethanol extract of *Ulmus pumila*.

Plant constituents	Ethanol extract
Alkaloids	−
Phenolics	+++
Flavonoids	+
Glycosides	++
Peptides	+
Steroids, terpenoids	+++
Organic acids	+

+++: strong; ++: moderate; +: poor; −: absent.
